# Physics, chemistry and biology of functional nanostructures III

**DOI:** 10.3762/bjnano.8.63

**Published:** 2017-03-09

**Authors:** Anatolie S Sidorenko

**Affiliations:** 1 D. Ghitsu Institute of Electronic Engineering and Nanotechnologies ASM, Academiei Str. 3/3, MD2028 Kishinev, Moldova

**Keywords:** functional nanostructures, nanomaterials, nanotechnology, sensors

Nanoscience and Nanotechnology became the trend of the 21st century, influencing many areas such as electronics, biology, medicine, aerospace, agriculture, and the engineering of novel materials. Recently a new aspect of nanoscience and nanotechnology appeared and has been rising rapidly ever since: functional nanostructures, intelligent nanostructures for targeted applications. In the present Thematic Series a bright spectrum of such targeted functional nanostructures is presented demonstrating the unique possibilities of engineering at the nanometer scale. The self-organization of nanoparticles, nanowires or nanotubes and the introduction of those structures into various materials made it possible to solve long-standing problems. Examples presented in this Thematic Series are:

- the problem of treatment of multidrug-resistant tuberculosis, which can be resolved using the invented nano-encapsulated medicines penetrating through the cell membrane of the tuberculosis bacilli [1],
- new applications of graphene-based nanostructures [2–4],
- smart nanoparticles with antitumor activity [5],
- photonic crystals and flexible membranes [6–7] for visualization devices and remote-readout strain gauges.

A lot of other unusual applications of functional nanostructures are presented in over 40 articles of this Thematic Series, giving the possibility to gain new knowledge about the rapidly developing area of nanoscience and nanotechnology.

The idea to this Thematic Series appeared during the international conference “NANO-2016: Ethical, Ecological and Social Problems of Nanoscience and Nanotechnologies”, which took place in May 2016 in Chisinau, Moldova. It presented new technological approaches for assembling of functional nanostructures and nanomaterials with special properties. As the guest editor of this Thematic Series I would like to thank all colleagues and highly experienced experts who submitted their manuscripts with novel results, new ideas, and technological approaches. I believe that this Thematic Series will attract attention of scientists, technologists, engineers and will find many readers. The professional and friendly editorial support by the Production Team of the Beilstein-Institut is greatly acknowledged.

Anatolie Sidorenko

Chisinau, January 2017

## References

[1] Ivancic A, Macaev F, Aksakal F, Boldescu V, Pogrebnoi S, Duca G (2016). Beilstein J Nanotechnol.

[2] Kim C-W, Dai M D, Eom K (2016). Beilstein J Nanotechnol.

[3] Sghaier T, Le Liepvre S, Fiorini C, Douillard L, Charra F (2016). Beilstein J Nanotechnol.

[4] Ortega-Amaya R, Matsumoto Y, Espinoza-Rivas A M, Pérez-Guzmán M A, Ortega-López M (2016). Beilstein J Nanotechnol.

[5] Arriortua O K, Garaio E, Herrero de la Parte B, Insausti M, Lezama L, Plazaola F, García J A, Aizpurua J M, Sagartzazu M, Irazola M (2016). Beilstein J Nanotechnol.

[6] Aluicio-Sarduy E, Callegari S, Figueroa del Valle D G, Desii A, Kriegel I, Scotognella F (2016). Beilstein J Nanotechnol.

[7] Karrock T, Paulsen M, Gerken M (2017). Beilstein J Nanotechnol.

